# The impact of individual-level heterogeneity on estimated infectious disease burden: a simulation study

**DOI:** 10.1186/s12963-016-0116-y

**Published:** 2016-12-08

**Authors:** Scott A. McDonald, Brecht Devleesschauwer, Jacco Wallinga

**Affiliations:** 1 Centre for Infectious Disease Control, National Institute for Public Health and the Environment, PO Box 1, 3720 BA Bilthoven, The Netherlands; 2 Department of Public Health and Surveillance, Scientific Institute of Public Health (WIV-ISP), Brussels, Belgium

**Keywords:** Infectious diseases, Heterogeneity, Disability-adjusted life years, Markov model

## Abstract

**Background:**

Disease burden is not evenly distributed within a population; this uneven distribution can be due to individual heterogeneity in progression rates between disease stages. Composite measures of disease burden that are based on disease progression models, such as the disability-adjusted life year (DALY), are widely used to quantify the current and future burden of infectious diseases. Our goal was to investigate to what extent ignoring the presence of heterogeneity could bias DALY computation.

**Methods:**

Simulations using individual-based models for hypothetical infectious diseases with short and long natural histories were run assuming either “population-averaged” progression probabilities between disease stages, or progression probabilities that were influenced by an *a priori* defined individual-level frailty (i.e., heterogeneity in disease risk) distribution, and DALYs were calculated.

**Results:**

Under the assumption of heterogeneity in transition rates and increasing frailty with age, the short natural history disease model predicted 14% fewer DALYs compared with the homogenous population assumption. Simulations of a long natural history disease indicated that assuming homogeneity in transition rates when heterogeneity was present could overestimate total DALYs, in the present case by 4% (95% quantile interval: 1–8%).

**Conclusions:**

The consequences of ignoring population heterogeneity should be considered when defining transition parameters for natural history models and when interpreting the resulting disease burden estimates.

**Electronic supplementary material:**

The online version of this article (doi:10.1186/s12963-016-0116-y) contains supplementary material, which is available to authorized users.

## Background

Disease burden, whether computed for infectious or for chronic diseases, is not evenly distributed within a population, or even among members of a particular stratum of the afflicted population. Relatively few afflicted individuals carry a disproportionate amount of the burden. This fact is obscured by the “population-averaged” approach to calculating and reporting standard epidemiological indicators, such as incidence, as well as composite measures of disease burden, such as disability-adjusted life years (DALYs). This individual-level heterogeneity in disease risk, often referred to as “frailty” [1–3], represents variation beyond that explained by known and measurable risk factors; such variation may be attributed to genetic, epigenetic, environmental, and/or stochastic factors. Unmeasured variation, when labeled as “randomness in degree of susceptibility,” has long been recognized as important for interpreting historical patterns in mortality and for improving the fit of demographic models [4], as well as for explaining age-dependent patterns of incidence for diseases such as testicular cancer [5]. Infectious diseases such as HIV, hepatitis C, and tuberculosis are also relevant candidates for such an analysis approach. However, although individual heterogeneity has been discussed in the context of health economic cost-effectiveness models [6, 7], and variability in transition rates has been modeled by specifying a distribution function fitted to clinical data [8], its impact has yet to be explicitly addressed in current disease burden estimation exercises.

The question to be addressed in the present paper is: Does ignoring individual-level heterogeneity in the rate of progressing from acute infection to more severe disease stages result in biased estimates of disease burden when using a disease progression pathway modeling approach to compute DALYs? Unobserved, unmeasured individual heterogeneity cannot be captured by covariates. Therefore, neither adjustment nor stratification are analysis options, and analytical or simulation methods are required to quantify the expected effects of ignoring unmeasured heterogeneity. The fundamental issue at stake concerns the impact of ignoring individual heterogeneity when ranking diseases according to their disease burden, which is a useful form of presentation for public health policymakers. If two diseases differ widely in terms of degree of individual heterogeneity – for instance if variation in individual-level susceptibility to rhinovirus infection differed greatly from susceptibility to *Campylobacter* – then their relative ranking may change substantially if individual heterogeneity is taken into account when computing DALYs. If such a ranking informs the prioritization of public health services, then appropriate computation of the absolute disease burden is vital. Thus, a first step towards understanding the effect of unmeasured heterogeneity on measures such as the DALY is to employ computational simulations to compare the expected disease burden in scenarios with and without such heterogeneity.

A potentially serious concern for the computation and interpretation of disease burden estimates relates to individual heterogeneity in rates of disease progression. Especially for chronic diseases, persons observed to be in the same disease stage may represent a wide range of individual disease progression rates, with consequences for the evaluation of interventions [9]. “Population-averaged” progression rates are typically employed in the disease progression pathway models that form the basis for pathogen-based disease burden estimation [10–12] However, a given patient population plausibly may contain relatively few fast-progressors, and many more slow-progressors; the oft-used population-averaged transition probability obscures the potential skewedness in the rate distribution, and disregards the extent of any variability as well as potential correlations between transition probabilities between successive health states. Although heterogeneity in infectivity or susceptibility to infection is also plausible, the current simulations do not consider this further, as transmission of infection is not modeled in the present study.

Below, we present simple natural history models (“outcome trees”) for two fictitious infectious diseases, “X_1_” and “*X*_2_,” and report the impact on estimated disease burden (in DALYs) when *a priori* assumed distributions of individual heterogeneity are incorporated into the model. The two hypothetical diseases are broadly representative of infectious diseases with short (e.g., Q fever) and long (e.g., hepatitis C virus infection) natural histories, but are not intended to correspond to specific diseases. Rather than implementing (possibly quite complex) disease progression pathways of actual infectious diseases, we simulate disease burden in simplified natural history models to facilitate interpretation of the results.

Our primary objective is to compare, for each of the two disease models, the disease burden for an infected cohort in which individual heterogeneity in progression probabilities between disease stages is present (heterogeneity variants), to the disease burden if this heterogeneity is ignored (no-heterogeneity variants). As a secondary objective, we investigate the impact of ignoring heterogeneity in disease progression rates on the disease burden averted due to a simulated public health intervention, namely high-coverage age-targeted vaccination.

## Methods

In the current study, we employ the term “frailty” more broadly than used in the statistical literature, where frailty refers to an unobserved random factor that modifies an individual’s hazard function. We use frailty to indicate an individual’s position within a population distribution of disease progression rates (specified as a Gamma distribution); individuals with higher frailty values progress more quickly than individuals with lower frailty values.

### Disease models

**Fig. 1:**
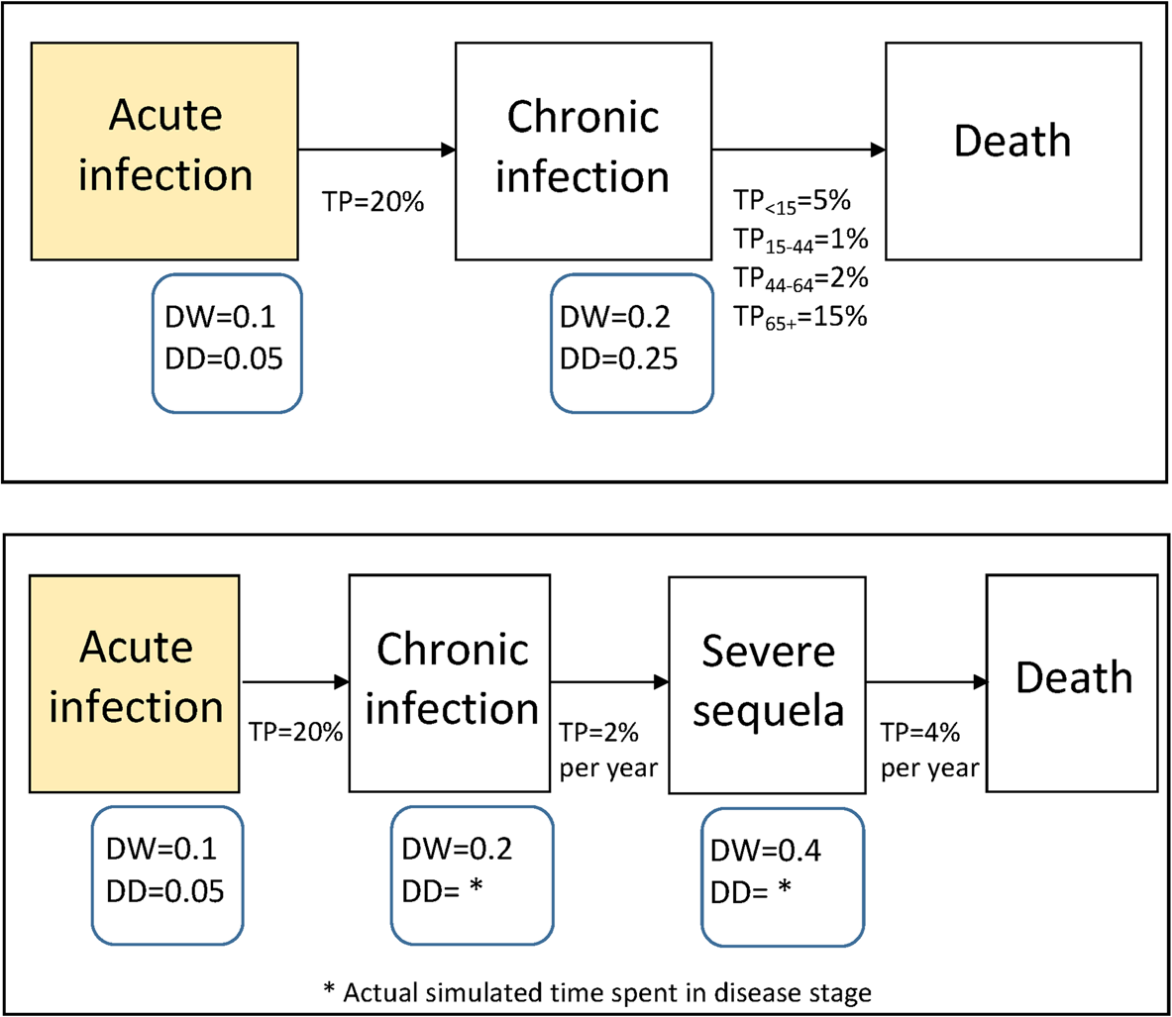
Outcome trees describing the natural histories of two hypothetical diseases, termed disease model X_1_: (three health outcomes: acute infection, chronic infection, and death following chronic infection) (*upper panel*), and disease model *X*_2_: (four health outcomes: acute infection, chronic infection, severe sequela, and death) (*lower panel*). DW = disability weight; DD = disability duration; TP = transition probability

Figure 1 shows two simplified outcome trees for the hypothetical diseases X_1_ and *X*_2_. Transition probabilities and other parameter values were not based on any existing disease progression pathway model, but were chosen for illustration purposes only. Both disease models consist of distinct stages, from acute infection through death. Disease model X_1_ is an example of a short natural history disease, in which progression from acute infection to chronic infection and/or complications and possible death occurs rapidly; for instance, within the first year following acute infection. In contrast, disease model *X*_2_ is an example of a disease with a long natural history, for which low annual transition probabilities between disease stages are specified. For the latter class of disease, slow progression means that the most severe disease stages may not be reached in the patient’s natural lifetime.

In disease model X_1_, acutely infected individuals develop chronic infection with an age-independent transition probability of 20% (for simulation purposes, this transition is assumed to effectively occur immediately). The transition probability for the risk of death following chronic infection is dependent on age, with case-fatality ratios of 5, 1, 2, and 15% specified for the <15, 15–44, 45–64, and 65+ years age-groups, respectively.

Disease model *X*_2_ simulates a disease with a long natural history. As for disease model X_1_, acutely infected individuals develop chronic infection with an age-independent probability of 20% (assumed to effectively occur immediately). Average progression from the chronic infection health outcome to severe sequela is assumed to be slow, with a transition probability of 2% per year. The annual probability of death following development of this sequela was set to 4%. Both of these transition probabilities are specified as age-independent. In this simulation, we needed to track individuals over time, and to simulate ageing of the acutely infected cohort. All individuals were assumed to die after reaching their 86th birthday, if they did not reach the death stage before this time.

To represent individual heterogeneity in the probability of transitioning from acute to chronic infection, from chronic infection to the severe sequela disease stage (model *X*_2_ only), and from severe sequela to death (model *X*_2_ only), we first assigned frailty values to each individual by random sampling from an age-independent frailty distribution. These frailty values are considered to be assigned at birth, and therefore did not change through an individual’s lifetime (see below). As a result, the more frail individuals were modeled to have higher transition probabilities for the relevant transitions, and the less frail to have lower transition probabilities. For model X_1_ only, individual heterogeneity in the progression from acute to chronic infection was assumed to be age-related, with mean frailty increasing with age. This leads to a stochastic tendency for developing chronic infection being more likely for older compared with younger individuals.

### Frailty distributions

**Fig. 2:**
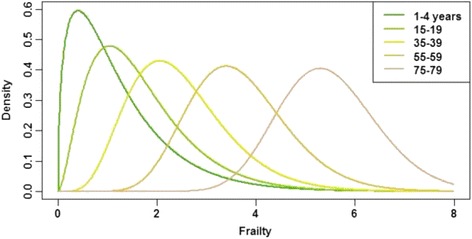
Frailty distributions assumed for the disease model X_1_ simulations (five age-groups only are shown). Gamma distributions were parameterised so that mean frailty increased exponentially with age-group, but variance was held constant at unity

Gamma distributions were defined to represent individual heterogeneity. For disease model X_1_, separate Gamma distributions were specified for each 5-year age-group (<1 years, 1–4, 5–9, 10–15, …, 80–84, 85+), with the mean of each frailty distribution assumed to be age-related, via an exponentially increasing (with age-group) shape parameter. The variance was kept constant across age-groups by adjusting the scale parameter accordingly. Figure 2 plots the resulting distributions for selected age-groups. For disease model *X*_2_, the age-independent Gamma distribution with unity mean and variance (i.e., shape and scale parameters set to 1) was used.

### Simulating disease progression and computing disease burden

**Fig. 3:**
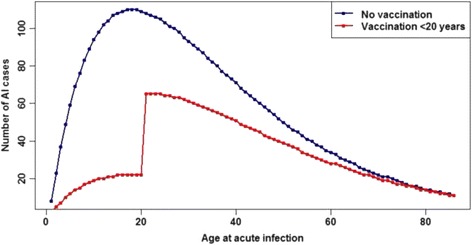
Age distribution of incident cases of acute infection used for both disease models X_1_ and *X*_2_, under the default scenario (X_1_ and *X*_2_ simulations) and the age-targeted vaccination scenario (*X*_2_ simulations only)

The assumed age distribution of incident (acute infection) cases is provided in Fig. 3. This distribution was defined using a Gamma(2,17.5) distribution, for which the mean is 35 years, but with a peak around 18–21 years. A total of 5000 incident cases was simulated.

We simulated disease progression in both disease models separately using an individual-based modeling approach, whereby each infected case was followed throughout disease progression, and the burden associated with each health outcome (and the sum over all outcomes) was computed using standard pathogen-based DALY methodology [10]. By using an individual-based modeling approach, we are thus able to account for the correlation in transition probabilities, which would be lost when using a population-averaged approach. In both disease models, all individuals are assumed to start in the acute infection disease stage. In disease model *X*_2_, identically sized cohorts of incident cases (*n* = 5000) entered the model each simulation year.

In the no-heterogeneity simulations, the expected YLD, YLL, and DALYs were computed from the expected number of cases progressing through an outcome tree defined by the transitional probabilities and Dutch male life expectancies for the year 2000 [13], and given assumed disability weights and durations (Fig. 1). Disease stage duration was truncated if the simulated individual reached their 86th birthday while in that disease stage (relevant for model *X*_2_ only). YLD and YLL measures were summed over all relevant health outcomes, with the DALY measure defined as the simple sum of YLD and YLL.

In the heterogeneity simulations, the central idea implemented was that the infected individuals who are most likely to transition to a subsequent disease stage, such as a complication or death, are those with the highest frailty. For these simulations, we first randomly sampled from the pre-defined frailty distributions (see below) and assigned frailty values to each individual. For disease model X_1_, the number of cases transitioning from acute to chronic infection was constrained to equal the expected cases (*N*) determined using the no-heterogeneity variant of the same model (to permit comparability between heterogeneity and no-heterogeneity variants). For disease model *X*_2_, the number of cases transitioning from a given health outcome to the subsequent health outcome in each simulation year was also constrained to equal the expected cases (*N*) based on the “population-averaged” transition probability. Stochastic sampling methods were used to determine which individuals transitioned from each health outcome. Specifically, *N* individuals were sampled without replacement, with the probability of being selected weighted according to each individual’s assigned frailty value. This procedure was then repeated for a total of 1000 times, with the median and 2.5 and 97.5% percentiles of the distributions of YLD, YLL, and DALYs reported.

### Sensitivity analyses

For disease model *X*_2_, the disease burden will be largely determined by the number of individuals who reach the death stage; the risk of death is dependent on the annual progression probabilities from the chronic infection and severe sequela stages. In sensitivity analysis, the effect of the initial choice of these parameter values on the simulated burden and on the overestimation of DALYs due to assuming population-averaged transition probabilities is explored. Additional file 1 reports the results of simultaneously varying the annual transition probabilities for the final two transitions in model *X*_2_ across a limited range. In a second sensitivity analysis involving model *X*_2_, two further frailty distributions are specified, and burden in DALYs compared with that obtained using the rightward-skewed distribution. In the first, skewedness was reversed (i.e., corresponding to a disease with few slow-progressors and many fast-progressors); in the second a peaked symmetrical distribution was tested (i.e., corresponding to a disease with equal (low) numbers of slow- and fast-progressors).

### Age-targeted vaccination scenario

We estimated the effect of a single simulated public health intervention, age-targeted vaccination with a simulated high coverage of 80%, and calculated DALYs averted. This was accomplished by retaining only 20% of the potential acute infection cases aged ≤19 years, very crudely simulating the effects of herd immunity on older age groups (see Fig. 2), and re-running the no-heterogeneity simulation. Then, the heterogeneity variant was run, to assess any change in the size of the vaccination effect. Note that a more accurate simulation of the impact of an age-targeted vaccination program would employ a dynamic modeling approach to simulate the time-dependent influence of herd immunity on successive birth cohorts entering the model.

Simulations were carried out in the R statistical programming environment, version 3.1.0 [14].

## Results

Table 1 compares the median YLD, YLL, and DALYs computed for the no-heterogeneity and heterogeneity variants of each of the two disease models considered. Note that the 95% quantile intervals for the heterogeneity model variants do not reflect uncertainty in incidence or model parameter values; rather, they only represent the effect of individual heterogeneity in progression probabilities on disease burden. In all cases, fewer DALYs are predicted under the heterogeneity variants than under the standard, no-heterogeneity variant. For both simple disease models, ignoring individual heterogeneity consistently overestimated disease burden. For disease model X_1_, although the number of individuals developing chronic infection was held constant across the no-heterogeneity and heterogeneity variants, the no-heterogeneity variant overestimated the total disease burden by a factor of 1.16 (95% interval: 1.11–1.22).

**Table 1:** Results of simulations using disease models X_1_ and *X*_2_, comparing the estimated disease burden between no-heterogeneity and heterogeneity variants. Results indicate the total burden for individuals acutely infected in simulation year 1, with 95% quantile intervals

Disease model [–Variant]	YLD	YLL (95% interval)	DALY (95% interval)	Overestimation of DALY (95% interval)
*X*_*1*_ *(3 health outcomes, 4 broad age-groups specified for transition from chronic infection to death)*				
– No heterogeneity	75	1243	1318	1.16 (1.11–1.22)
– Heterogeneity^a^	75	1060 (1007–1117)	1135 (1082–1192	–
*X*_*2*_ *(4 health outcomes)*				
– No heterogeneity	10750	10210 (9380–11090)	20960 (20140–21740)	1.04 (1.01–1.08)
– Heterogeneity^b^	11010	9074 (8411–9887)	20090 (19440–20780)	–

*Note*: ^a^Gamma distributions, with mean increasing with age. ^b^Age-independent, sampled from Gamma(1,1). Overestimation of DALY is with respect to heterogeneity model variant

**Fig. 4:**
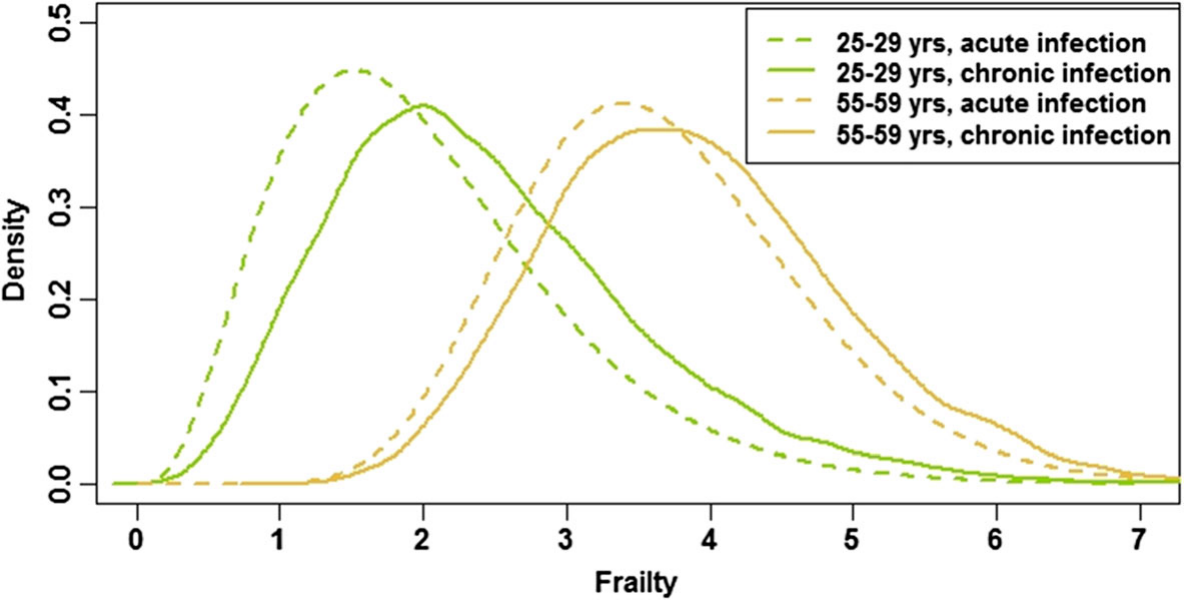
Frailty distributions of individuals in the acute infection and chronic infection disease stages in disease model X_1_. Two selected age-groups are plotted, before (*dashed line*) and after (*solid line*) the transition from acute to chronic infection

In Fig. 4 (disease model X_1_, selected age-groups shown), the expected rightward shift in frailty distribution before and after transitioning from the acute to the chronic infection stage is illustrated. The frailest individuals, in general and within a given age-group, are more likely to progress to a more advanced disease stage. The mean frailty values for individuals within the acute and chronic infection disease stages was 2.39 and 3.17, respectively.

For disease model *X*_2_, in which a disease with a long natural history was simulated via specification of annual transition probabilities, overestimation of total disease burden by the no-heterogeneity variant was by a factor of 1.04 (95% interval: 1.01–1.08) (Table 1). This difference was driven by YLL (overestimated by a factor of 1.12), as YLD was actually larger for the heterogeneity variant. The “trade-off” between YLD and YLL is due to a greater proportion of infected persons spending more time in the chronic infection and severe sequela stages in the heterogeneity compared with the no-heterogeneity variant (leading to a higher YLD), and the corresponding fewer deaths in the former variant (leading to lower YLL).

**Fig. 5:**
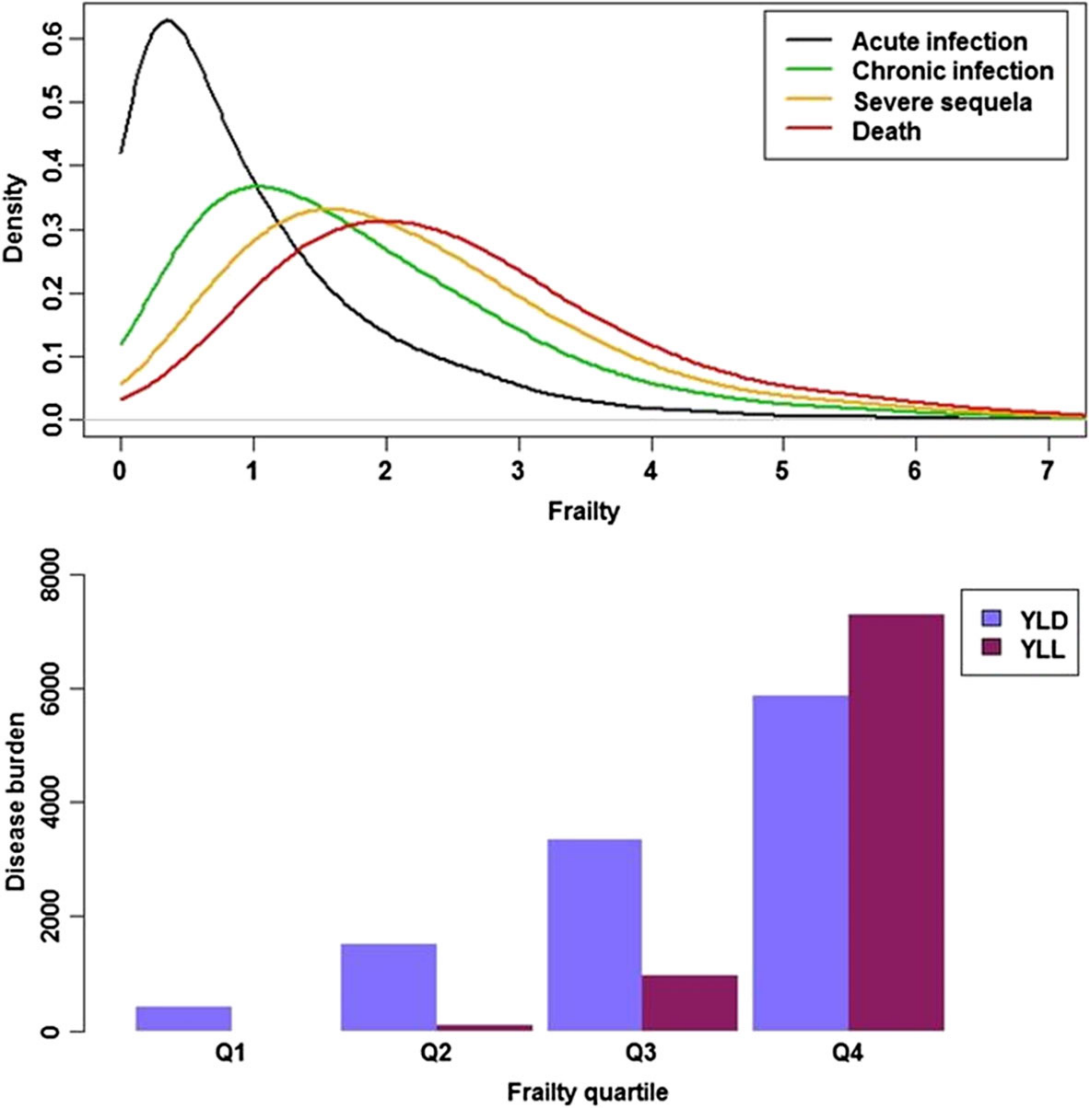
Frailty distributions of the members of the infected cohort entering each disease stage, for disease model *X*_2_ (*upper panel*). Disease burden plotted separately as YLD and YLL, as function of frailty quartile (Q1 = first quartile, etc.), for disease model *X*_2_ (*lower panel*)

The expected rightward shift in frailty distribution with disease stage is illustrated by Fig. 5a. Figure 5b confirms the larger burden borne by the frailest individuals; individuals with the lowest 25% frailty experience no disease-related mortality, and little morbidity (low YLD), whereas those in the top 25% frailty quartile have the largest YLD and YLL. The proportion of total disease burden comprised by YLL also increases with frailty quartile, surpassing YLD for persons with the highest 25% frailty.

**Fig. 6:**
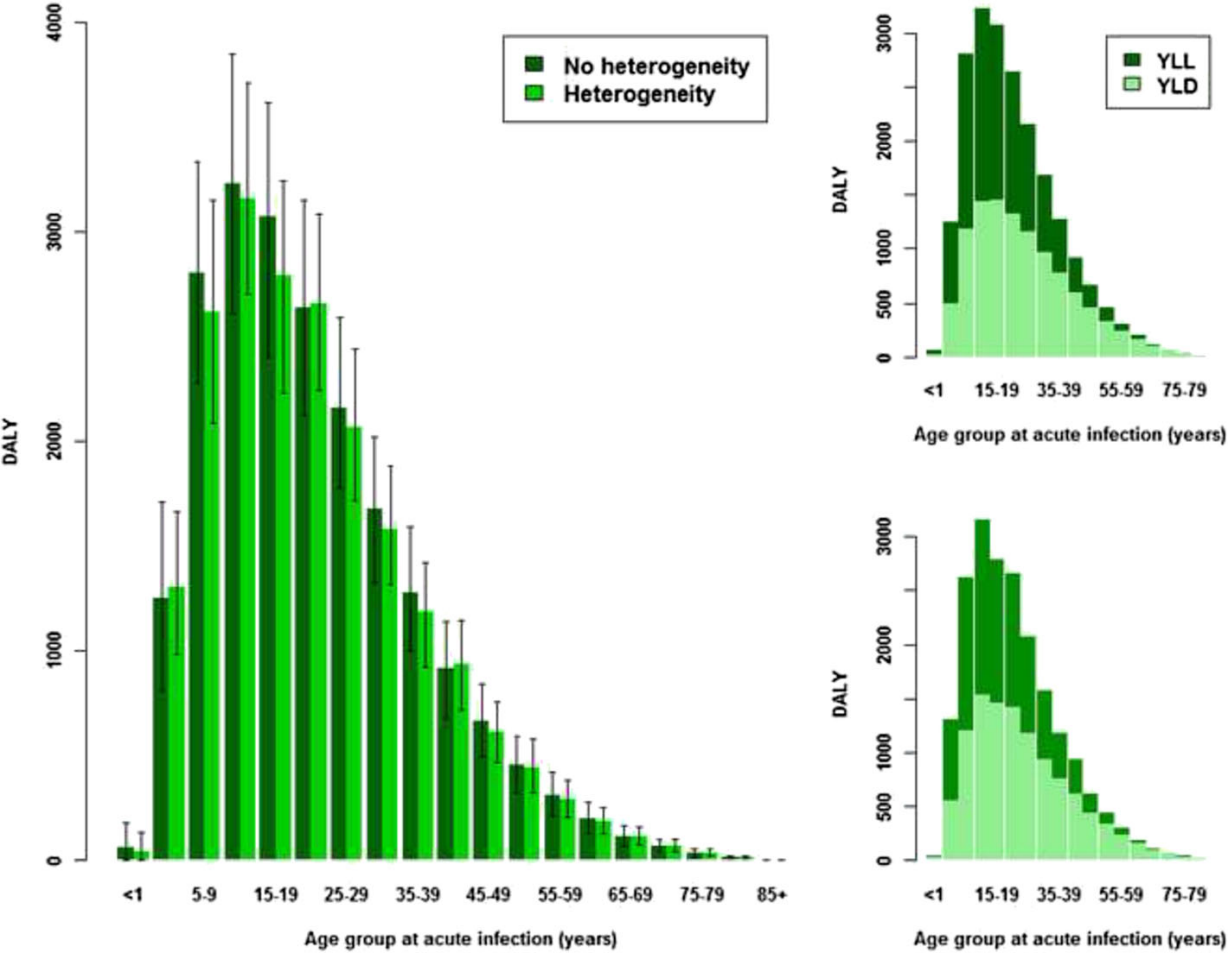
Comparison of estimated disease burden over age-group at acute infection for disease model *X*_2_, with and without heterogeneity in disease progression rates (*main panel*): heterogeneity leads to an overall lower burden. The two smaller plots show disease burden by age-group split into YLD and YLL, for the no-heterogeneity (*upper right panel*) and heterogeneity (*lower right panel*) model variants. Capped lines indicated 95% quantile intervals

In Fig. 6, the burden associated with age-group at acute infection is shown as estimated using the two disease model *X*_2_ variants. The lower burden for the heterogeneity compared with the no-heterogeneity variant is localized to those individuals acutely infected between the ages of 5 and 39 years (Fig. 6.)

The results of the first sensitivity analysis indicated that the values initially chosen for the progression probabilities from the chronic infection and severe sequela stages resulted in a disease burden overestimation factor on the high end for the range of parameter values investigated (Additional file 1). This factor tended to increase as either annual probability increased (leading to more mortality at a younger age), with a range of 1.01 to 1.08. In the second sensitivity analysis, leftward-skewed and peaked symmetrical frailty distributions were investigated; the resulting greater DALYs compared with the rightward-skewed distribution (main analysis) obtained with both alternatives lends support to our central finding from disease model *X*_2_: burden is lower when there are a relatively greater number of slow- than fast-progressors, because of the smaller number of premature deaths.

The simulation of high-coverage age-targeted (<20 years) vaccination suggested that this strategy – preventing 2232 acute infections – would reduce the disease burden for the conventional (no heterogeneity) disease model *X*_2_ by 56% (Table 2). 11,680 DALYs would be expected to be averted under the assumption of population homogeneity in progression rate. For the heterogeneity variant of disease model *X*_2_, the expected impact of vaccination was nearly identical (55%): an average of 11,140 DALYs would be averted under the assumption of individual heterogeneity in transition probabilities.

**Table 2:** Simulation results: estimated burden under vaccination and no-vaccination scenarios using disease model *X*_2_. Results indicate the total burden for individuals acutely infected in simulation year 1, with 95% quantile intervals

Model variant [–Vacc. scenario]	Acute cases	YLD	YLL (95% interval)	DALY (95% interval)	Burden averted DALY (%)
*X*_*2*_ *No heterogeneity*					
– No vaccination	5000	10750	10210 (9380–11090)	20960 (20140–21740)	–
– Vaccination <20 year	2768	5212	4066 (3537–4635)	9280 (8765–9770)	11680 (55.7%)
*X*_*2*_ *Heterogeneity* ^a^					
– No vaccination	5000	11010	9074 (8411–9887)	20090 (19440–20780)	–
– Vaccination <20 year	2768	5252	3693 (3259–4145)	8945 (8536–9374)	11140 (55.0%)

*Note*: ^a^Individual-level heterogeneity was specified as age-independent; frailty values were sampled from Gamma(1,1)

## Discussion

To what extent does individual heterogeneity in disease progression rates affect the computation of composite disease burden measures, such as the DALY? Our principal finding is the following: if the degree of individual heterogeneity that we simulated in transition probability distributions mimics the extent of unmeasured heterogeneity in the population, then ignoring this heterogeneity can result in inflated disease burden estimates. In the case of disease model X_1_, the simulated dependence of mean frailty on age in the heterogeneity variant is responsible for the lower disease burden compared with the no-heterogeneity variant. With a skewed frailty distribution, a minority of patients die young, with the majority living to an older age, compared with application of a population-averaged transition probability. This resulted in a smaller YLL – and a consequent 14% lower total disease burden – being estimated for the heterogeneity than for the no-heterogeneity variant.

For disease model *X*_2_, in which frailty distributions were specified as age-independent (i.e., assumed fixed at the age of acute infection), the 4% lower burden estimated for the heterogeneity variant is due entirely to the simulated heterogeneity in disease progression rates. This is because even though the most frail individuals progress the most rapidly through the disease course, and therefore have a higher probability of developing severe sequelae and dying at a younger age, on average disease progression is slower than if heterogeneity is ignored. Due to the skewedness of the frailty distribution, only a minority of patients are fast progressors; for the majority of patients, disease progression is slow, and the severe disease stages, if experienced during their lifetime, are reached at a later age.

The lower estimated burden for the heterogeneity variant is therefore due to fewer members of an acutely infected cohort reaching the age at which severe sequela or death due to the disease can occur (and thus resulting in a lower YLL); however, individuals in this variant tended to spend longer in chronic infection and severe sequela stages compared with the no-heterogeneity model, which resulted in a higher YLD. Despite this YLD/YLL “trade-off,” there is an overall reduced burden in the heterogeneity variant, most apparent in the younger age-groups (5- to 39-year-olds) (Fig. 6) due to their lower risk of dying from the disease before reaching their life expectancy.

It might be argued that the X_1_ simulations only demonstrate that availability of age-dependent transition probabilities in place of a single age-independent transition probability is vital, if the incident case population covers a wide age range and the risk of developing a complication or dying is greater for older than for younger patients. Because in disease model X_1_ the frailest patients are the most likely to transition, assuming increasing mean frailty with age effectively translates to a statistical preference for older patients transitioning to chronic infection before younger patients. Disease model *X*_2_ – which explicitly simulates aging of an acutely infected cohort simultaneously with progression through the various disease stages – illustrates that the assumption of age-dependent mean frailty is unnecessary for longer natural history diseases.

For disease model X_1_, ignoring age-dependent heterogeneity leads to overestimation of disease burden; but is it plausible that mean frailty would increase with age? Although there are health states for which the young are at the greatest risk, frailty in general may be roughly monotonic with age. Cumulative occasions of ill health from birth (the “insult accumulation” model [4]) would lead to an individual’s frailty – and so his/her susceptibility to disease progression and/or death – also increasing with age. Also, declining mortality rates or increasing life expectancy over a period of time would give rise to an age-related frailty effect [15]. Finally, in the case of infectious diseases, immunosenescence (age-associated decline in immune function) could contribute to an increasing susceptibility to development of complications and death [16].

For diseases with a long natural history, as exemplified by disease model *X*_2_, comparison of simulations assuming age-independent heterogeneity with the no-heterogeneity variant suggested that ignoring a plausible degree of individual heterogeneity in disease progression when computing DALYs would lead to a 4% overestimation of the total expected burden among a cohort of acutely infected persons. Although the magnitude of DALY overestimation is small, mortality burden was overestimated by 14%. If the prioritization of public health resources are informed by a ranking of diseases according to overall burden or mortality burden, differential overestimation (i.e., individual heterogeneity affecting burden estimates for a subset of the ranked diseases) may have important consequences. In addition, if such a disease model is used to project the impact of a prevention initiative such as age-targeted vaccination, heterogeneity could influence the size and/or direction of the intervention effect, and therefore investigation of the impact of homogeneity assumptions is important for decision-making [7]. However, in our *X*_2_ simulation incorporating individual heterogeneity, the vaccination effect size (on DALYs) was virtually identical to the projected effect size for the no-heterogeneity variant.

Application of the concepts investigated in the current paper to epidemiological studies in which disease burden is estimated is relatively unexplored. Estimation of the extent of unmeasured heterogeneity can in principle be done by fitting a statistical model to a longitudinal dataset that records disease state transition times among a cohort of infected individuals, but certain assumptions are required [2], and interpretation must be made with caution. If a variance parameter can be validly estimated for a given transition, then the calculation of DALYs, for instance, could incorporate this variability, by specifying a relevant distribution in place of a single “population-averaged” transition probability [8].

## Conclusions

In conclusion, the current findings corroborate what has been reported regarding the influence of heterogeneity in Markov models for cost-effectiveness [6, 7]: ignoring heterogeneity can produce either optimistic or pessimistic cost-effectiveness ratios, with consequent impact on the use of such ratios for the planning of interventions. The heterogeneity issue could apply to every transition in a Markov model used for disease burden calculation; therefore, when selecting parameters for this type of model and interpreting the resulting burden estimates, the analyst should consider the consequences of assuming that the population within each health outcome is homogenous with regard to transition rates. If this homogeneity assumption cannot be made, an individual-based modeling approach is the most appropriate solution.

## Additional file

Additional file 1: Sensitivity analyses. (DOCX 86 kb)
