# Supplementary material for: The impact of individual-level heterogeneity on estimated infectious disease burden: a simulation study
Source: Popul Health Metr. 2016 Dec 8;14:47. doi: 10.1186/s12963-016-0116-y (PMC5146833; doi:10.1186/s12963-016-0116-y)
Supplement: Additional file 1: — Sensitivity analyses. (DOCX 86 kb) [file 12963_2016_116_MOESM1_ESM.docx]

**Additional File 1**: Sensitivity analyses

To accompany the article:

*Title*: The impact of individual-level heterogeneity on estimated infectious disease burden: a simulation study

*Authors*: Scott A. McDonald, Brecht Devleesschauwer, Jacco Wallinga

**Sensitivity analysis 1:** Varying annual transition probability parameters

The table below shows the sensitivity of estimated DALYs for no-heterogeneity and heterogeneity variants of model X_2_, according to variation in two parameters: the population-averaged annual transition probabilities for progression from the chronic infection to the severe sequela stage, and for progression from the severe sequela stage to death. Shaded cells indicate the parameter values and the results reported in the main text.

| **Annual transition probability  parameter values** | **No-heterogeneity DALY (95% interval)** | **Heterogeneity**  **DALY (95% interval)** | **Overestimation  of DALY  (95% interval)** |
| --- | --- | --- | --- |
| **CI Sequela = 1%/yr** | | | |
| Sequela Death = 2%/yr | 15660 (15040-16280) | 15530 (14930-16150) | 1.01 (0.97-1.05) |
| Sequela Death = 4%/yr | 16780 (16060-17480) | 16620 (16070-17240) | 1.01 (0.97-1.04) |
| Sequela Death = 6%/yr | 17520 (16720-18280) | 17260 (16570-17940) | 1.01 (0.98-1.06) |
| **CI Sequela = 2%/year** | | | |
| Sequela Death = 2%/yr | 18950 (18130-19660) | 18490 (17850-19160) | 1.03 (0.99-1.06) |
| Sequela Death = 4%/yr | 20960 (20140-21740) | 20090 (19440- 20780) | 1.04 (1.01-1.08) |
| Sequela Death = 6%/yr | 22140 (21250-22980) | 21310 (20490-22030) | 1.04 (1.01-1.08) |
| **CI Sequela = 3%/year** | | | |
| Sequela Death = 2%/yr | 21400 (20680-22110) | 20560 (19920-21270) | 1.04 (1.01-1.07) |
| Sequela Death = 4%/yr | 23924 (23035-24794) | 22790 (22030-23540) | 1.05 (1.02-1.09) |
| Sequela Death = 6%/yr | 25310 (24440-26200) | 24120 (23380-24950) | 1.05 (1.02-1.08) |
| **CI Sequela = 4%/year** |  |  |  |
| Sequela Death = 2%/yr | 23070 (22320-24060) | 21730 (21000-22550) | 1.06 (1.02-1.10) |
| Sequela Death = 4%/yr | 26020 (25140-26970) | 24290 (23470-25080) | 1.07  (1.04-1.11) |
| Sequela Death = 6%/yr | 27720 (26950-28750) | 25770 (25000-26660) | 1.08 (1.04-1.11) |

*Note*. CI = chronic infection; DALY = disability-adjusted life year

**Sensitivity analysis 2:** Varying the shape of the frailty distribution


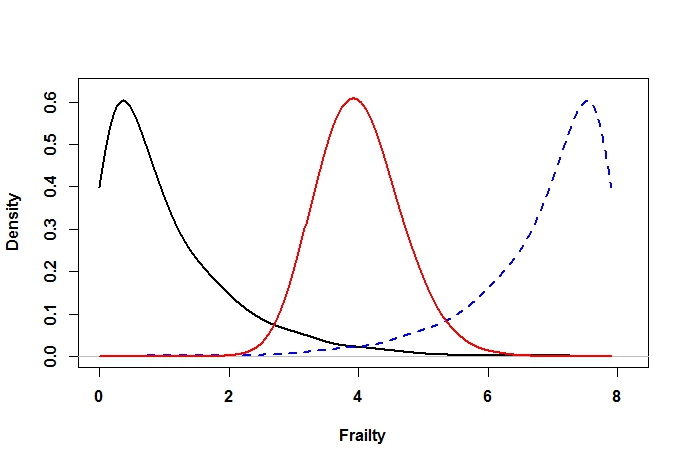
In this supplementary analysis conducted using disease model X_2_, the effect on estimated DALYs of specifying (i) a frailty distribution skewed towards fast progressors (blue dashed line in below figure), and (ii) a centered frailty distribution (red line; *Gamma*(40,0.1), which is approximately Gaussian) are compared with the rightward-skewed frailty distribution used in the primary simulations (black line), is examined. The below table shows the sensitivity of estimated DALYs for no-heterogeneity and heterogeneity variants of model X_2_, according to the shape manipulation.

| **Disease model  variant** | **YLD** | **YLL  (95% interval)** | **DALY (95% interval)** | **Overestimation of DALY (95% interval)** |
| --- | --- | --- | --- | --- |
| No-heterogeneity | 10750 | 10210 (9380–11090) | 20960 (20140–21740) | 1.04 (1.01–1.08) |
| Heterogeneity (rightward-skewed)^a^ | 11010 | 9074 (8411–9887) | 20090 (19440-20780) | – |
| Heterogeneity (centred)^b^ | 10770 | 10160 (9283-11020) | 20930 (20130-21710) | 1.04 (1.01-1.08) |
| Heterogeneity (leftward-skewed)^c^ | 10740 | 10190 (9284-11010) | 20920 (20160-21660) | 1.04 (1.01-1.08) |

*Note*. ^a^ Sampled from Gamma(1,1); burden values identical to Table 1. ^b^ Sampled from Gamma(40,0.1). ^c^ Sampled from Gamma(1,1), then reversed by subtracting from the maximum sampled value. Overestimation of DALY is with respect to the heterogeneity (rightward-skewed) model variant.
